# Non-Invasive Molecular Survey of Sarcoptic Mange in Wildlife: Diagnostic Performance in Wolf Faecal Samples Evaluated by Multi-Event Capture–Recapture Models

**DOI:** 10.3390/pathogens10020243

**Published:** 2021-02-20

**Authors:** Julieta Rousseau, Mónia Nakamura, Helena Rio-Maior, Francisco Álvares, Rémi Choquet, Luís Madeira de Carvalho, Raquel Godinho, Nuno Santos

**Affiliations:** 1 CIISA—Centro de Investigação Interdisciplinar em Sanidade Animal, Faculty of Veterinary Medicine, University of Lisbon, Avenida da Universidade Técnica, 1300-477 Lisbon, Portugal; 2 CIBIO/InBIO—Centro de Investigação em Biodiversidade e Recursos Genéticos, Universidade do Porto, Campus de Vairão, 4485-661 Vairão, Portugal; moniayui@gmail.com (M.N.); helenariomaior@gmail.com (H.R.-M.); falvares@cibio.up.pt (F.Á.); rgodinho@cibio.up.pt (R.G.); nuno.santos@cibio.up.pt (N.S.); 3 Department of Biology, Faculty of Sciences, University of Porto, 4169-007 Porto, Portugal; 4 CEFE—Centre d’Écologie Fonctionnelle et Évolutive, UMR 5175, CNRS, University of Montpellier, 1919 Route de Mende, CEDEX 5, FR-34293 Montpellier, France; Remi.CHOQUET@cefe.cnrs.fr

**Keywords:** *Canis lupus*, *Vulpes vulpes*, *Sarcoptes scabiei*, PCR, serology, epidemiology, Iberian Peninsula

## Abstract

Sarcoptic mange is globally enzootic, and non-invasive methods with high diagnostic specificity for its surveillance in wildlife are lacking. We describe the molecular detection of *Sarcoptes scabiei* in non-invasively collected faecal samples, targeting the 16S rDNA gene. We applied this method to 843 Iberian wolf *Canis lupus signatus* faecal samples collected in north-western Portugal (2006–2018). We further integrated this with serological data (61 samples from wolf and 20 from red fox *Vulpes vulpes*, 1997–2019) in multi-event capture–recapture models. The mean predicted prevalence by the molecular analysis of wolf faecal samples from 2006–2018 was 7.2% (CI_95_ 5.0–9.4%; range: 2.6–11.7%), highest in 2009. The mean predicted seroprevalence in wolves was 24.5% (CI_95_ 18.5–30.6%; range: 13.0–55.0%), peaking in 2006–2009. Multi-event capture–recapture models estimated 100% diagnostic specificity and moderate diagnostic sensitivity (30.0%, CI_95_ 14.0–53.0%) for the molecular method. Mange-infected individually identified wolves showed a tendency for higher mortality versus uninfected wolves (ΔMortality 0.150, CI_95_ −0.165–0.458). Long-term serology data highlights the endemicity of sarcoptic mange in wild canids but uncovers multi-year epidemics. This study developed and evaluated a novel method for surveying sarcoptic mange in wildlife populations by the molecular detection of *S. scabiei* in faecal samples, which stands out for its high specificity and non-invasive character.

## 1. Introduction

Sarcoptic mange is a highly contagious and globally widespread skin disease caused by the burrowing mite *Sarcoptes scabiei*, affecting more than 100 wild and domestic mammal species [1]. Its transmission occurs mainly by intra- or interspecific direct contact, but indirect transmission can also occur if mites contaminate the environment, such as dens [2,3]. Sarcoptic mange causes severe pruritus, accompanied by erythematous eruptions, papules, alopecia, and crusts [4]. As the disease progresses, it can give rise to a complex cascade of interacting physiological and behavioural effects on the host, which can lead to death [5]. These effects are related to compromised thermoregulatory capacity, increased metabolic rates, and altered activity patterns [5,6].

Sarcoptic mange is enzootic in several wildlife populations throughout the world, but may become epizootic [7,8]. Epizootics can occur due to the introduction of a new virulent strain of *S*. *scabiei*, increased pathogenicity of an already existing strain, and/or increased susceptibility of the host population [9]. The impact of the disease is potentially more severe in small, genetically compromised, or fragmented populations, mediated by demographic stochasticity, weakened immune responses, and lack of metapopulation dynamics [1,10,11].

Surveillance of sarcoptic mange in wildlife can be accomplished by invasive or non-invasive methods. In non-invasive sampling, the source sample is left behind by the animal and can be recovered without having to capture or disturb it [12]. To date, mange surveillance tends to rely on invasive methods, such as the detection of *S. scabiei* in skin samples by molecular methods, microscopy, or the recovery of live mites [13,14], or by serology resorting to blood samples, body fluids, or lung tissue [15,16,17]. These methods are challenging due to the elusiveness of wildlife and require the capture or death of individuals, raising welfare issues [18,19]. The availability of non-invasive surveillance methods is thus of utmost relevance [20]. Camera-trapping is a non-invasive method that has been providing insights into the epidemiology of alopecic lesions in wildlife populations [13,21]. However, it is constrained by low diagnostic specificity, because it only detects mange-compatible dermatological lesions, without confirmation of the aetiology [1,21].

The genetic analysis of non-invasive samples, such as faeces, can be a valuable tool for the surveillance of mange [20]. The associated pruritus causes the affected animals to spend much time grooming, potentially ingesting mites and passing them in their faeces [20]. Besides sarcoptic mange, the same occurs in notoedric mange, caused by the sarcoptiform mite *Notoedres* sp., which is most common in felids [22]. Taking advantage of this, Stephenson et al. [20] developed a PCR assay targeting the internal transcribed spacer 2 (ITS-2) gene for the detection of *Notoedres cati* DNA in faecal samples of bobcats *Lynx rufus*, providing insights into the distribution of the disease in northern California, U.S.A. However, PCR assays targeting ITS-2 and the cytochrome c oxidase subunit 1 (COX-1) genes were unsuccessful in detecting the DNA of *S. scabiei* in faecal samples of black bears *Ursus americanus* [23]. 

Longitudinal surveillance of wildlife diseases can be presented as capture–recapture (CR) data, which correspond to an individual’s history of encounters [24], whenever samples can be assigned to individually identified animals. Capture–recapture data can be analysed through multi-event CR models, which consider states (usually “infected”/“uninfected”/“dead”) and observations generated from the underlying state of an individual, while accounting for the uncertainty in state assignment and imperfect detection [25]. These states, often not observable in the field, are linked by probabilistic matrices to observable events [25]. At each encounter occasion, an event is observed and recorded in an encounter history. Multi-event CR models were initially developed for ecological studies; however, they have proven to be powerful tools to study infectious and parasitic diseases in wildlife, allowing the estimation of epidemiological parameters related to infection rates and survival [26,27]. These models can be used to evaluate the performance of diagnostic tests, because they estimate the uncertainty in state assignment, which corresponds to the diagnostic sensitivity and specificity of the test.

Regarding wild canids, the study of sarcoptic mange by conventional methods is hampered by their elusive behaviour and low population densities [20,28]. The red fox *Vulpes vulpes* is often considered as a reservoir host [8,13], and sarcoptic mange has caused local declines of this species in Europe [7,29]. While wolves, *Canis lupus*, seem to usually mount an effective immune response to sarcoptic mange [4,13], the disease caused average mortality estimated at 27–34% in the Midwestern U.S.A. [1]. Such estimates are lacking for Europe [6], where wolf populations are more fragmented than in North America [28]. Particularly, the Iberian Peninsula maintains an isolated, stable to slightly increasing population of >2000 Iberian wolves *Canis lupus signatus* [28], where 20% seroprevalence and mortality caused by sarcoptic mange has been reported [13,30]. 

Given the potential impact of sarcoptic mange on populations of wild canids and the lack of specific non-invasive surveillance techniques, this study aims to: (i) optimize a molecular method for the surveillance of sarcoptic mange in non-invasive faecal samples of wolves; (ii) use multi-event capture–recapture models, integrating serology and molecular data, to evaluate the performance of the non-invasive method and estimate epidemiological parameters of sarcoptic mange in this wolf population.

## 2. Results

### 2.1. Non-Invasive Molecular Method

A 16S rDNA PCR fragment of the same size of the positive control (132 bp) was amplified in 39/843 faecal samples, while in another 32 samples the amplified fragment was 1–2 bp larger or smaller than the positive control. We assumed these differences as the result of polymorphisms (insertions or deletions) in the hypervariable regions of the 16S rDNA. We thus classified the 71 samples as positive to *S. scabiei* DNA. In 48 of the 71 (68%) samples, the results of the two replicas were concordant. 

The generalized linear mixed model (GLMM) showed non-significant differences in prevalence between sexes (*p* = 0.405) and sampling at wolf homesites or in transects (*p* = 0.471) (Table 1). 

**Figure 1:**
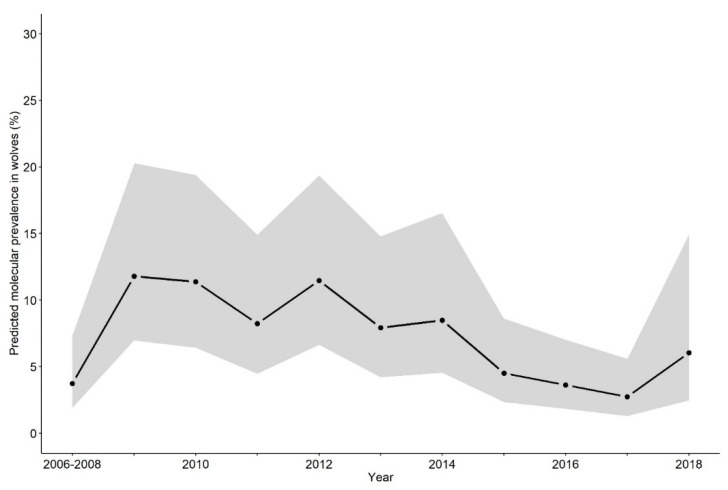
Predicted prevalence of sarcoptic mange in wolves by the non-invasive molecular method, from 2006 to 2018. An 80% confidence interval of the fixed and random effects from the binomial generalized linear mixed model is shaded in grey.

The overall prevalence of sarcoptic mange in the non-invasive wolf faecal samples, estimated by GLMM from 2006 to 2018, was 7.2% (CI_95_ 5.0–9.4%). The predicted prevalence was highest in 2009 (11.7% CI_95_ 5.1–26.9%) and lowest in 2017 (2.6% CI_95_ 0.9–8.3%) (Figure 1).

### 2.2. Serology

The GLMM showed non-significant tendencies for higher seroprevalence in foxes compared to wolves (*p* = 0.437) and lower in lung tissue extract (LTE) compared to serum (*p* = 0.076) (Table 2). 

**Figure 2:**
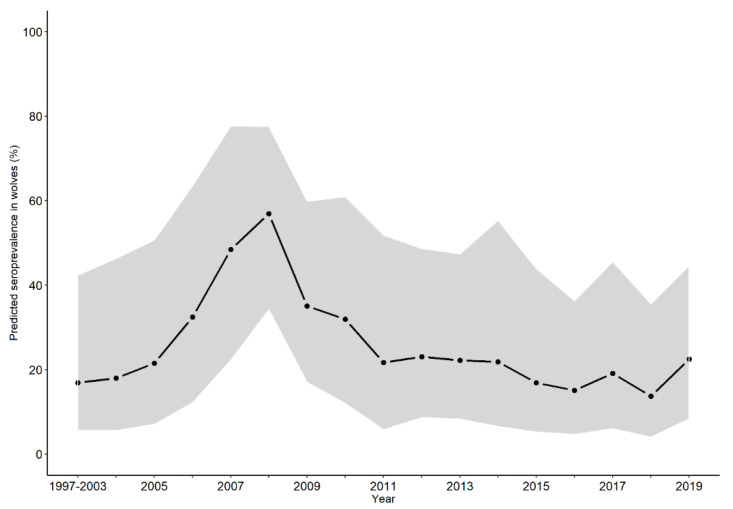
Predicted seroprevalence of sarcoptic mange in wolves considering serum as the test matrix, from 1997 to 2019. The 80% confidence interval of the fixed and random effects from the binomial generalized linear mixed model is shaded in grey.

The overall predicted seroprevalence of sarcoptic mange in wolves from 1997 to 2019 was 24.5% (CI_95_ 18.5–30.6%). The predicted seroprevalence in wolves ranged from 13.0% (CI_95_ 2.4–46.4%) in 2018, to 55.0% (CI_95_ 23.0–84.2%) in 2008, and highlights an outbreak peaking between 2006 and 2009 (Figure 2). 

### 2.3. Multi-Event Capture–Recapture Models

The most supported model (Akaike information criterion corrected for small sample size (AICc) = 1115.46, Model 1, Supplementary Table S1) assumed no difference in mortality between mange-infected and uninfected wolves. This model estimated that 30.0% (CI_95_ 14.0–53.0%) of the infected wolves would test positive, which corresponds to the diagnostic sensitivity of the non-invasive molecular method. It also estimated that 100% of the uninfected wolves test negative, which corresponds to the diagnostic specificity of the non-invasive molecular method. This last estimate was consistent across models, even in those where this parameter was estimated instead of fixed (Model 4, Supplementary Table S1). 

**Figure 3:**
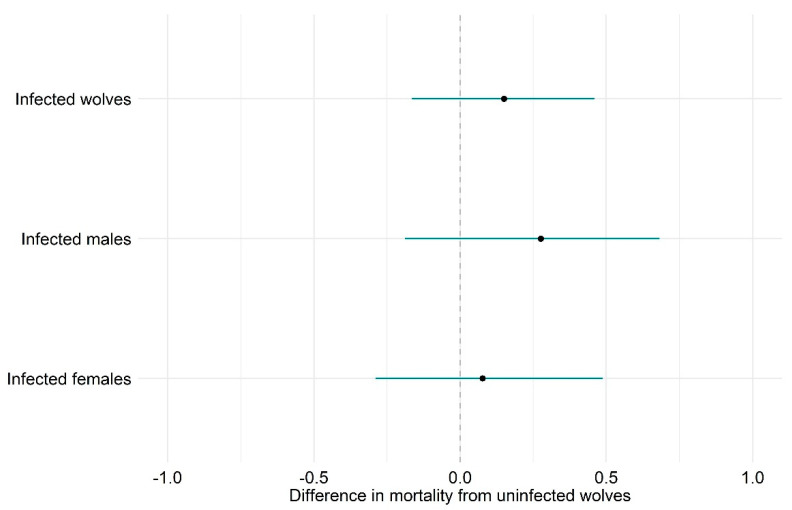
Difference in the estimated mortality between sarcoptic mange-infected and uninfected wolves. Estimates from the multi-event capture–recapture models 2 and 3 (Supplementary Table S1). The 95% confidence intervals of the difference in the mortality estimates are shown as horizontal error bars.

While the most supported model does not include an effect of sarcoptic mange on survival, there is good support for the model including such an effect (ΔAICc = 0.94, model 2, Supplementary Table S1). Model 2 estimated the mortality of individually identified infected wolves to be higher by 0.150 (CI_95_ −0.165–0.458) than for uninfected wolves (Figure 3). Furthermore, there is moderate support for a differential effect of sarcoptic mange on survival between sexes; model 3 (ΔAICc = 2.02, Supplementary Table S1) estimated the mortality of individually identified infected male wolves to be higher by 0.199 (CI_95_ −0.377–0.671) than for infected females (Figure 3). 

## 3. Discussion

Sarcoptic mange in wildlife populations is notoriously difficult to monitor because of the elusive nature of the hosts and the difficulty in obtaining invasive biological samples, which also raises animal welfare concerns [13,18,21]. Here, we describe a novel method for the surveillance of sarcoptic mange in wildlife, relying on PCR assays applied to non-invasively collected faecal samples. The present study is, to the best of our knowledge, the first to successfully detect *S. scabiei* DNA in wildlife using non-invasive samples. This method has implications for wildlife management because it allows disease surveillance avoiding capture and immobilization, which require considerable time and resources and have implications for animal welfare [18,19]. It also allows individual genetic identification and facilitates achieving larger sample sizes and repeated sampling over time, essential for longitudinal epidemiological studies.

However, non-invasive genetic samples have potential drawbacks such as the possibility of false-negative results due to the poor quality of the DNA in aged samples, the presence of PCR inhibitors, inconsistent grooming and ingestion of mites, the dilution of low numbers of ingested mites in large volumes of faeces, or intermittent mite excretion [20,23]. These issues likely contribute to the moderate diagnostic sensitivity achieved by the method (30.0%), as estimated by multi-event CR models. The study by Stephenson et al. [20] on notoedric mange in bobcats estimated a sensitivity of 52.6%, somewhat higher than in our study on sarcoptic mange in wolves. The number of mites ingested is probably influenced by the different pathology of mange in the two species. Sarcoptic mange in wolves is usually characterized by low parasite loads [4], however notoedric mange in bobcats presents numerous parasites [22]. Additionally, grooming behaviour is more pronounced in felids than in canids [31]. We thus hypothesize that the diagnostic sensitivity of molecular methods based on faecal samples depends on the pathology of the disease in the host species, as well as on its grooming behaviour. Furthermore, the poor quality of the DNA in aged faecal samples (the majority of our sample) likely contributed to the rate of false negatives, although we were able to amplify *Sarcoptes* spp. DNA from samples without individual genetic identification (i.e., presumably with more degraded DNA).

While multi-event CR models provided relevant information on the diagnostic performance of the non-invasive method, more research is warranted. Particularly relevant would be to compare the estimates of the diagnostic sensitivity and specificity with those obtained from animals with known infections status and varying parasite loads, although these are difficult to obtain in wild species. False-positive results arising from pseudo-parasitism from the consumption of mange-infected prey or allogrooming of infected conspecifics could be expectable [32]. However, our results suggest that such events are infrequent; in all the most supported multi-event CR models, the probability of amplifying *S. scabiei* DNA from faecal samples of uninfected wolves was estimated as null. 

The predicted prevalence of *S. scabiei* DNA in faecal samples of wolves in our study region was 7.2% from 2006–2018. No differences were found in prevalence by sex, as previously reported in Iberian wolves [13]. Significant temporal variation in prevalence was identified, with a maximum predicted by GLMM in 2009 (11.7%) and minimum in 2017 (2.6%). This temporal trend is mostly concordant with the pattern we found using serological methods.

The detection of antibodies against *S. scabiei* in wolves and foxes using a commercial enzyme-linked immunosorbent assay (ELISA) kit for dogs is warranted by the antigenic similarity between varieties of *S. scabiei* [23,33,34]. Although the test employed uses HRP-conjugated anti-dog antibodies, the immunological similarity between canid species makes it possible to detect antibodies against *S. scabiei* in fox and wolf samples, as shown in other studies [15,23]. 

The overall seroprevalence estimated by GLMM from 1997 to 2019 was 24.5%, peaking during 2006–2009, up to 55.0% in 2008. Interestingly, an outbreak of sarcoptic mange in wolves and red foxes was also recorded in Asturias, northern Spain (~200 km from our study area) in 2007–2008 [13]. In the Iberian Peninsula, in 2004–2008, seroprevalence in Asturias averaged 20.5%, similar to our estimate [13]. The seroprevalence detected in our study was higher than that reported for Scandinavian wolves (10.1%) during 1998–2013, which was hypothesized to be related to higher densities of wild carnivores and greater contact of wolves with domestic animals in the Iberian Peninsula [35].

Finite mixture models allow characterizing the distributions of the seropositive and seronegative subgroups within bimodal datasets [36], thus being an alternative tool to estimate the probability of each sample being positive or negative to serological tests [37]. These models have been increasingly used to evaluate the performance of diagnostic tests in the absence of reference tests in humans [38,39,40] and livestock [41,42]. They are particularly relevant in epidemiology studies in wildlife, where reference tests or reference infected/uninfected animals are seldom available [37]. The multi-species nature and limited size of our dataset, including wolves and red foxes sampled post-mortem, might have affected the diagnostic performance of the serological test. However, we must emphasize the use of lung tissue extract, with the advantage of the ease of collection (simple, cheap and without the need for specialized personnel), the possibility of freezing cadavers or organs until the test is performed and the serological evaluation of animals submitted to necropsy, as being particularly useful when studying elusive wild species [38,39,40]. The representativeness of our serological study could be affected by the limited size and opportunistic nature of the sample collection. We strived to control for these confounding factors by using mixed models with “biological matrix” and “species” as independent variables and present the seroprevalence data predicted by the model accounting for these confounding factors (Figure 2). Nevertheless, this limitation of our study is evident in the wide confidence intervals of the predicted seroprevalence.

The integration of *S. scabiei* DNA detection in individually identified faecal samples with invasive serological data in multi-event CR models is a powerful tool to estimate epidemiological parameters. Our data support that wolves infected with sarcoptic mange might have higher mortality compared to uninfected wolves (0.15). The mortality was also elevated (0.27–0.34) in mange-infected wolves in North America [1], mediated by pack size, because diseased wolves could obtain nutritional support from the pack’s predation [32]. Furthermore, individual wolves’ genomic variation was shown to be negatively correlated with sarcoptic mange severity [43].

Interestingly, the excess mortality of mange-infected wolves was estimated in our study to be larger for males than for females. Although there is a general trend for higher parasite loads in males than females in many wildlife species, as assessed by prevalence and intensity of infection, evidence for differential mortality between sexes is scant [44,45]. Sex was not a significant determinant of sarcoptic mange severity in Yellowstone wolves [43]. The apparently elevated susceptibility of male Iberian wolves to sarcoptic mange could be due to immunological, hormonal, or behavioural sexual differences [44]. 

In the Iberian Peninsula, despite wolf mortality from sarcoptic mange being occasionally recorded [30], it was suggested that the disease had a limited demographic effect on wolf populations [13]. Although the confidence interval of our estimates does not exclude null differences in mortality (Figure 3), it suggests higher mortality in Iberian wolves infected by sarcoptic mange, particularly in males, which may raise conservation issues and requires further research. 

The Iberian wolf population is the largest in western Europe, and it diverged from other European wolves approximately ten thousand years ago [46]. Most of Europe’s wolf populations have been increasing, however the Iberian population is, in general, stable [28]. Inhabiting a largely human-dominated landscape, such as our study area [47], frequent contact with humans and domestic animals may contribute to the occurrence of sarcoptic mange outbreaks [1,35]. Additionally, the Iberian wolf population is highly structured, presents low connectivity [48], and exhibits low genetic diversity in the major histocompatibility complex class II locus [49]. This low diversity reported at a locus involved in the immune response to extracellular parasites [50] could lead to increased susceptibility to sarcoptic mange. 

We highlight the particular case of the small and threatened subpopulation of Iberian wolves located south of the Douro river in Portugal, consisting of fewer than 10 packs showing reproductive instability, low genetic diversity, and isolation [48,51], and one of the few in Europe considered on the verge of extinction [52]. The impact of infectious and parasitic diseases, with the foremost example of sarcoptic mange, in isolated populations can lead to population declines and even local extinctions [7,29,32]. It is therefore essential to implement disease surveillance schemes, preferentially using non-invasive methods such as the one we have established and proposed here as a novel diagnostic tool for this zoonotic disease in wildlife. 

## 4. Materials and Methods

### 4.1. Study Area

This study was conducted in an ~8000 km^2^ region located in north-western Portugal (south-western Europe), comprising a mountainous area up to 1430 m above sea level (a.s.l.) with a temperate Atlantic–Mediterranean climate characterised by hot summers and rainy winters, with annual average precipitation of 1200 mm with little snow (<30 days/year of snow cover) and average annual temperature of 14 °C [53,54]. The study area encompasses a human-dominated landscape [47] inhabited by a largely stable population of 15–20 wolf packs, which prey mostly on livestock [51,55]. The red fox is an abundant and widespread wild canid in the study area [56]. 

### 4.2. Molecular Screening of Faecal Samples

The Iberian wolf faecal samples were collected between 2006 and 2018 in a subset of the area surveyed by serology (~2000 km^2^ inhabited by 5–8 wolf packs) [47]. DNA was available for a total of 843 scats collected using preventive measures to avoid sample contamination and stored at room temperature in sterile tubes in 96% ethanol [57]. Faecal samples were systematically collected either along transects (*n* = 557) or at wolf homesites (*n* = 286), including both fresh and aged samples [47,57]. All samples were previously confirmed as belonging to wolves by genetic analysis, and information on the sex and individual identification (genotype) was available for most of the samples (*n* = 445, from 219 individuals), following the procedures described in Nakamura et al. [57]. Briefly, DNA was extracted from the scat outer layer following the protocol of Frantz et al. [58] using the GuSCN/silica approach [59]. As a final step, DNA was purified using Microcon YM-30 columns (Millipore, Billerica, MA, USA) [60]. Negative controls were included throughout the process to monitor for potential DNA contamination, and all pre-PCR procedures were performed in laboratories dedicated to low-quality DNA samples [57]. Until further processing, DNA was stored at −20 °C.

In the present study, DNA was extracted from a skin sample of a *S. scabiei*-infected red fox (as confirmed by microscopy) and used as a positive control in the PCRs. DNA was extracted using the EasySpin Genomic DNA Tissue Kit (Citomed, Lisbon, Portugal), following the manufacturer’s instructions. The presence of a 132 bp fragment from the 16S rDNA mitochondrial gene of *S. scabiei* was tested in the 843 non-invasive samples using primers SSUDF and SSUDR, as described by Angelone-Alasaad et al. [14]. An M13-tail fluorescence labelling protocol was implemented to allow size screening in an automated sequencer [61]. The PCR reaction was prepared with a final volume of 10.5 µL, consisting of 5 µL of MyTaq HS Mix (Bioline, London, UK), 0.04 µL of primer forward, 0.4 µL of primer reverse, 0.4 µL of fluorescent M13-tail, and 2.5 µL of water. Primers were used at 10 µM. PCRs were prepared in plates, each including negative and positive controls, in rooms dedicated to low-quality DNA. Each sample was assayed twice to increase the probability of amplification. Samples were considered positive if one of the replicas amplified the target DNA sequence.

DNA amplification was performed in a T100 thermocycler (Bio-Rad, Hercules, CA, USA) following the protocol: initial denaturation of 10 min at 95 °C and 45 cycles of 30 s at 95 °C, 45 s at 53 °C, and 20 s at 72 °C, with a final extension of 10 min at 72 °C. The amplified products were separated by capillary electrophoresis in an ABI3130xl Genetic Analyser (Applied Biosystems, Foster City, CA, USA), and fragment size scoring was performed against the GeneScan 500 LIZ molecular size standard (Applied Biosystems) using GeneMapper 5 (Applied Biosystems). Results were checked manually.

### 4.3. Collection of Invasive Samples for Serology

Between 1997 and 2019, whole blood samples were opportunistically collected from Iberian wolves (*n* = 22) and red foxes (*n* = 5) whenever they were captured for scientific purposes, as described in Santos et al. [19]. Briefly, wild canids were captured with Belisle leg-hold snares (Edouard Belisle, Saint Veronique, QC, Canada) and chemically immobilised with an intramuscular injection of a mixture of ketamine (4.71 ± 1.17 mg/kg) (Imalgene, Boehringer Ingelheim, Lyon, France) and medetomidine (0.10 ± 0.03 mg/kg) (Domitor, Boehringer Ingelheim, Lyon, France). Whole blood was collected by venepuncture of the cephalic or saphenous veins, and the serum obtained by centrifugation at 1430× *g* for 10 min was stored at −20 °C until its use. The chemical immobilization was reversed with an intramuscular injection of atipamezole (0.40 ± 0.01 mg/kg) (Revertor, Boehringer Ingelheim, Lyon, France) [19]. Trapping was conducted under permits issued by the Portuguese nature conservation authority (*Instituto da Conservação da Natureza e das Florestas:* 338/2007/CAPT, 258/2008/CAPT, 286/2008/CAPT, 260/2009/CAPT, 332/2010/MANU, 333/2010/CAPT, 336/2010/MANU, 26/2012/MANU, and 72/2014/CAPT) and according to Portuguese (Decreto-Lei 113/2013) and European legislation (Directive 2010/63/EU) on animal experimentation and international wildlife standards [62,63].

In the same period, lung samples were collected upon standard necropsy of opportunistically-found dead wolves (*n* = 39) and red foxes (*n* = 15) and stored at −20 °C until processing. Lung tissue extract (LTE) was obtained following the protocol by Ferroglio et al. [64] with minor adaptations. Briefly, ~1 g of lung tissue was cut into 4–5 pieces and placed in a Falcon tube, where 1 mL of phosphate-buffered saline was added and shaken manually for 4 min. Samples were then centrifuged at 800× *g* for 10 min and the supernatant was collected and stored at −20 °C until use.

### 4.4. Serology

Antibodies against *S. scabiei* antigens were detected with a commercial indirect ELISA kit (*Sarcoptes*-ELISA 2001 Dog Kit, AFOSA GmbH, Germany) following the manufacturer’s instructions with minor adaptations. The antibody concentration in LTE was shown in other species to be 1–3-fold lower compared to serum [64,65]. To minimize this expected difference between the two biological matrices used in this study, sera were tested at a dilution of 1:100 in sample dilution solution, according to the manufacturer’s instructions, and LTE at 1:50. Sera, LTE samples, and controls (dog sera provided with the kit) were tested in duplicate. The test result (TR) was calculated according to the equation:

(1) $$\mathrm{TR}=\frac{\mathrm{ODsample}-\mathrm{ODnegative}\_\mathrm{control}}{\mathrm{ODpositive}\_\mathrm{control}-\mathrm{ODnegative}\_\mathrm{control}}\times 100$$

The ELISA was applied to types of samples (serum/LTE) for which it was not originally validated; therefore, it was essential to estimate the cut-off for positivity. The cut-off was estimated by finite mixture models, which enable characterizing of the distributions of the subgroups (seropositive and seronegative in this study) within bimodal datasets [36]. These models are powerful tools in the scope of probabilistic diagnostics of serological tests in the absence of reference tests [37,39]. 

Finite mixture modelling of the TR data was performed separately for sera and LTE, because differences in antibody concentration may occur between these biological matrices [40]. A non-parametric stochastic model for independent data was implemented, which does not assume any type of distribution for the seropositive and seronegative subsets of the dataset [36]. The distribution of the seropositive and seronegative subsets is thus solely derived from the data. The probability of each sample belonging to the seropositive or seronegative subsets was estimated from 2000 model iterations, and each sample was assigned to one of those subsets when the estimated probability was >95%. Finite mixture models were implemented using the package “mixtools” [36] in R. 

### 4.5. Statistical Analysis

Binomial generalized linear mixed models (GLMM) with a logit link were used to identify the variables related to serology and to non-invasive molecular results, as well as to correct for potential confounding factors related to the sampling process. The categorical independent variables included in the serology GLMM were the test matrix (serum/LTE) and species (wolf/fox), as fixed effects, and year (1997–2019), as a random effect. The reference classes were set based on their sample size and proportion of positives: serum and wolf. 

In the non-invasive molecular GLMM, the categorical independent variables were the type of sample collection (transect/homesites) and sex, as fixed effects, and year (2006–2018), as a random effect. The reference classes were samples collected in transects, and female, for the abovementioned reasons.

Some of the years were pooled (1997–2003 in the serology GLMM and 2006–2008 in the molecular GLMM) to achieve adequate sample sizes in each of the models (Table 1 and Table 2). Correlation between fixed effects was always <0.600. The serological and molecular prevalence of mange in wolves for each year, the overall prevalence, and the corresponding 80% and 95% confidence intervals for the random effects were predicted from the GLMM using the package “merTools” [66]. *p*-values were calculated following Satterthwaite’s degrees of freedom method using the package “lmerTest” [67]. All statistical analyses were performed in R 3.6.1 [68].

### 4.6. Multi-Event Capture–Recapture Models

Multi-event CR models were applied to a subset of the non-invasive molecular data for which individual identification was already available (445 samples, assigned to 219 individual wolves between 2006 and 2018). The following states were considered in the model: wolves infected with *S. scabiei* (M+), uninfected wolves (M−), and dead wolves (D). In any sampling occasion (year), an individual wolf may be alive in classes M+ or M− or may be dead. In each sampling occasion, the possible observations were: “individual wolf not detected”, “individual wolf detected and *S. scabiei* PCR-negative”, “individual wolf detected and *S. scabiei* PCR-positive”, or “individual wolf detected but not tested for *S. scabiei*”.

Models were implemented with the software E-SURGE [24], which uses a maximum likelihood approach to estimate the parameters. The model included the following matrices: initial state, survival, transitions between M+ and M− conditional on survival, detection, probability of being tested, and uncertainty in state assignment (Supplementary File S1). An EM (20) + Quasi-Newton nonlinear maximum likelihood solver was used to obtain the maximum likelihood estimator, and 50 model runs using a different set of random initial values were applied to avoid local minima. 

A set of candidate models were defined, incorporating biologically relevant combinations of effects on survival (constant, sarcoptic mange, and sex effects). The standardized annual predicted seroprevalence of sarcoptic mange estimated from the serology GLMM was included as a temporal covariate to estimate the transition between the M- and M+ states (equivalent to the incidence of the infection). For the events, the detection probability at the first encounter was fixed at 1 because the encounter history is conditional on being caught in the first period, and the following detection probabilities depend on the state and the time occasion. The probability of being assigned the observation “individual wolf detected but not tested for *S. scabiei*” was fixed at the proportion of individually identified samples that were not tested for *S. scabiei* DNA (0.247). Regarding the uncertainty in state assignment, the probability of a non-infected wolf testing positive was consistently estimated as close to null, and was thus fixed at 0 in models 1–3 and 5 (Supplementary File S1).

No multi-event goodness-of-fit test exists; therefore, we applied a single-state goodness-of-fit testing approach implemented in U-CARE [69]. Test 3.SR indicated significant transience effects (*χ*^2^ = 20.91, *p* = 0.034, 11 df). Transience can be defined as individuals captured for the first time having a lower probability of being re-captured, as compared to individuals that had been captured previously [70]. Survival was thus estimated in all models separately for wolves captured once or more than once [70]. Different models were selected under an information–theoretical approach by their Akaike information criterion corrected for small sample size (AICc) [71].

## 5. Conclusions

We describe a novel non-invasive method for monitoring sarcoptic mange in wildlife, based on the detection of *S. scabiei* DNA in faecal samples. Although the method was developed and validated using wolf samples, it should be useful in other wildlife hosts, and stands out for its high specificity and non-invasive character. The application of multi-event CR models to datasets with individually identified samples was fundamental for estimating epidemiological parameters in mange-infected wolves and for evaluating the performance of the new surveillance method.

## Figures and Tables

**Table 1:** Results of the binomial generalized linear model of non-invasive molecular data in wolves.

Variable	Samples (*n*)	β	Standard Error (β)	*z*-Score	*p*-Value
Fixed effects					
Intercept		−2.710	0.399	−6.801	<0.001
Sex					
Male	237	0.309	0.371	0.832	0.405
Type of sampling					
Homesites	286	−0.317	0.440	−0.721	0.471
Random effect					
Variance	0.519				
Standard deviation	0.721				
Years (N)	11				
Samples (*n*)	442				

*n* sample size; N total number of units.

**Table 2:** Results of the binomial generalized linear mixed model of serology data in wolves and red foxes.

Variable	Samples (*n*)	β	Standard Error (β)	*z*-Score	*p*-Value
Fixed effects					
Intercept		−1.199	0.600	−1.999	0.046
Test matrix					
Lung tissue extract	54	−1.238	0.698	−1.775	0.076
Species					
Red fox	20	0.582	0.749	0.777	0.437
Random effect					
Variance	1.023				
Standard deviation	1.012				
Years (N)	17				
Samples (*n*)	80				

*n* sample size; N total number of units.

## Data Availability

The data that support the findings of this study are available from the corresponding author upon reasonable request.

## Supplementary Materials

The following are available online at https://www.mdpi.com/2076-0817/10/2/243/s1. File S1: Methodological approach for the multi-event capture–recapture models; Table S1: Parametrizations of the most supported model and models with ΔAICc < 4. Differences to the most supported model highlighted in bold.

## References

[1] Niedringhaus K.D., Brown J.D., Sweeley K.M., Yabsley M.J. (2019). A review of sarcoptic mange in North American wildlife. Int. J. Parasitol. Parasites Wildl..

[2] Kołodziej-Sobocińska M., Zalewski A., Kowalczyk R. (2014). Sarcoptic mange vulnerability in carnivores of the Białowieża Primeval Forest, Poland: Underlying determinant factors. Ecol. Res..

[3] Montecino-Latorre D., Cypher B.L., Rudd J.L., Clifford D.L., Mazet J.A.K., Foley J.E. (2019). Assessing the role of dens in the spread, establishment and persistence of sarcoptic mange in an endangered canid. Epidemics.

[4] Oleaga A., Casais R., Prieto J.M., Gortázar C., Balseiro A. (2012). Comparative pathological and immunohistochemical features of sarcoptic mange in five sympatric wildlife species in Northern Spain. Eur. J. Wildl. Res..

[5] Martin A.M., Fraser T.A., Lesku J.A., Simpson K., Roberts G.L., Garvey J., Polkinghorne A., Burridge C.P., Carver S. (2018). The cascading pathogenic consequences of *Sarcoptes scabiei* infection that manifest in host disease. R. Soc. Open Sci..

[6] Astorga F., Carver S., Almberg E.S., Sousa G.R., Wingfield K., Niedringhaus K.D., Van Wick P., Rossi L., Xie Y., Cross P. (2018). International meeting on sarcoptic mange in wildlife, June 2018, Blacksburg, Virginia, USA. Parasites Vectors.

[7] Soulsbury C.D., Iossa G., Baker P.J., Cole N.C., Funk S.M., Harris S. (2007). The impact of sarcoptic mange *Sarcoptes scabiei* on the British fox *Vulpes vulpes* population. Mamm. Rev..

[8] Davidson R.K., Bornstein S., Handeland K. (2008). Long-term study of *Sarcoptes scabiei* infection in Norwegian red foxes (*Vulpes vulpes*) indicating host/parasite adaptation. Vet. Parasitol..

[9] Pence D.B., Windberg L.A., Pence B.C., Sprowls R. (1983). The epizootiology and pathology of sarcoptic mange in coyotes, *Canis latrans*, from South Texas. J. Parasitol..

[10] Pence D.B., Ueckermann E. (2002). Sarcoptic mange in wildlife. OIE Rev. Sci. Tech..

[11] Benson J.F., Mahoney P.J., Vickers T.W., Sikich J.A., Beier P., Riley S.P.D., Ernest H.B., Boyce W.M. (2019). Extinction vortex dynamics of top predators isolated by urbanization. Ecol. Appl..

[12] Taberlet P., Waits L.P., Luikart G. (1999). Noninvasive genetic sampling: Look before you leap. Trends Ecol. Evol..

[13] Oleaga A., Casais R., Balseiro A., Espí A., Llaneza L., Hartasánchez A., Gortázar C. (2011). New techniques for an old disease: Sarcoptic mange in the Iberian wolf. Vet. Parasitol..

[14] Angelone-Alasaad S., Molinar Min A.R., Pasquetti M., Alagaili A.N., D’Amelio S., Berrilli F., Obanda V., Gebely M.A., Soriguer R.C., Rossi L. (2015). Universal conventional and real-time PCR diagnosis tools for *Sarcoptes scabiei*. Parasites Vectors.

[15] Bornstein S., Frössling J., Näslund K., Zakrisson G., Mörner T. (2006). Evaluation of a serological test (indirect ELISA) for the diagnosis of sarcoptic mange in red foxes (*Vulpes vulpes*). Vet. Dermatol..

[16] Jakubek E.B., Mattsson R., Mörner T., Mattsson J.G., Gavier-Widén D. (2012). Potential application of serological tests on fluids from carcasses: Detection of antibodies against *Toxoplasma gondii* and *Sarcoptes scabiei* in red foxes (*Vulpes vulpes*). Acta Vet. Scand..

[17] Haas C., Rossi S., Meier R., Ryser-Degiorgis M.-P. (2015). Evaluation of a commercial ELISA for the detection of antibodies to *Sarcoptes scabiei* in wild boar (*Sus scrofa*). J. Wildl. Dis..

[18] Lindsjö J., Fahlman Å., Törnqvist E. (2016). Animal welfare from mouse to moose—Implementing the principles of the 3Rs in wildlife research. J. Wildl. Dis..

[19] Santos N., Rio-Maior H., Nakamura M., Roque S., Brandão R., Álvares F. (2017). Characterization and minimization of the stress response to trapping in free-ranging wolves (*Canis lupus*): Insights from physiology and behavior. Stress.

[20] Stephenson N., Clifford D., Worth S.J., Serieys L.E.K., Foley J. (2013). Development and validation of a fecal PCR assay for *Notoedres cati* and application to notoedric mange cases in bobcats (*Lynx rufus*) in Northern California, USA. J. Wildl. Dis..

[21] Carricondo-Sanchez D., Odden M., Linnell J.D.C., Odden J. (2017). The range of the mange: Spatiotemporal patterns of sarcoptic mange in red foxes (*Vulpes vulpes*) as revealed by camera trapping. PLoS ONE.

[22] Foley J., Serieys L.E.K., Stephenson N., Riley S., Foley C., Jennings M., Wengert G., Vickers W., Boydston E., Lyren L. (2016). A synthetic review of *Notoedres* species mites and mange. Parasitology.

[23] Peltier S.K., Brown J.D., Ternent M.A., Fenton H., Niedringhaus K.D., Yabsley M.J. (2018). Assays for detection and identification of the causative agent of mange in free-ranging black bears (*Ursus americanus*). J. Wildl. Dis..

[24] Choquet R., Rouan L., Pradel R., Thomson D.L., Cooch E.G., Conroy M.J. (2009). Program E-SURGE: A software application for fitting multievent models. Modeling Demographic Processes in Marked Populations. Environmental and Ecological Statistics Series.

[25] Pradel R. (2005). Multievent: An extension of multistate capture-recapture models to uncertain states. Biometrics.

[26] Lachish S., Knowles S.C.L., Alves R., Wood M.J., Sheldon B.C. (2011). Infection dynamics of endemic malaria in a wild bird population: Parasite species-dependent drivers of spatial and temporal variation in transmission rates. J. Anim. Ecol..

[27] Chambert T., Staszewski V., Lobato E., Choquet R., Carrie C., Mccoy K.D., Tveraa T., Boulinier T. (2012). Exposure of black-legged kittiwakes to Lyme disease spirochetes: Dynamics of the immune status of adult hosts and effects on their survival. J. Anim. Ecol..

[28] Chapron G., Kaczensky P., Linnell J.D.C., von Arx M., Huber D., Andrén H., López-bao J.V., Adamec M., Álvares F., Anders O. (2014). Recovery of large carnivores in Europe’s modern human-dominated landscapes. Science.

[29] Forchhammer M.C., Asferg T. (2000). Invading parasites cause a structural shift in red fox dynamics. Proc. R. Soc. B Biol. Sci..

[30] Domínguez G., Espí A., Prieto J.M., De La Torre J.A. (2008). Sarcoptic mange in Iberian wolves (*Canis lupus signatus*) in northern Spain. Vet. Rec..

[31] Read J., Gigliotti F., Darby S., Lapidge S. (2014). Dying to be clean: Pen trials of novel cat and fox control devices. Int. J. Pest Manag..

[32] Almberg E.S., Cross P.C., Dobson A.P., Smith D.W., Metz M.C., Stahler D.R., Hudson P.J. (2015). Social living mitigates the costs of a chronic illness in a cooperative carnivore. Ecol. Lett..

[33] Lower K.S., Medleau L.M., Hnilica K., Bigler B. (2001). Evaluation of an enzyme-linked immunosorbant assay (ELISA) for the serological diagnosis of sarcoptic mange in dogs. Vet. Dermatol..

[34] Arlian L.G., Morgan M.S. (2017). A review of *Sarcoptes scabiei*: Past, present and future. Parasites Vectors.

[35] Fuchs B., Zimmermann B., Wabakken P., Bornstein S., Månsson J., Evans A.L., Liberg O., Sand H., Kindberg J., Ågren E.O. (2016). Sarcoptic mange in the Scandinavian wolf *Canis lupus* population. BMC Vet. Res..

[36] Benaglia T., Chauveau D., Hunter D.R., Young D.S. (2009). Mixtools: An R package for analyzing finite mixture models. J. Stat. Softw..

[37] Peel A.J., McKinley T.J., Baker K.S., Barr J.A., Crameri G., Hayman D.T.S., Feng Y.R., Broder C.C., Wang L.-F., Cunningham A.A. (2013). Use of cross-reactive serological assays for detecting novel pathogens in wildlife: Assessing an appropriate cutoff for henipavirus assays in African bats. J. Virol. Methods.

[38] Ades A.E., Price M.J., Kounali D., Akande V.A., Wills G.S., McClure M.O., Muir P., Horner P.J. (2017). Proportion of tubal factor infertility due to *Chlamydia*: Finite mixture modeling of serum antibody titers. Am. J. Epidemiol..

[39] Migchelsen S.J., Martin D.L., Southisombath K., Turyaguma P., Heggen A., Rubangakene P.P., Joof H., Makalo P., Cooley G., Gwyn S. (2017). Defining seropositivity thresholds for use in trachoma elimination studies. PLoS Negl. Trop. Dis..

[40] Seck M.C., Badiane A.S., Thwing J., Moss D., Fall F.B., Gomis J.F., Deme A.B., Diongue K., Sy M., Mbaye A. (2019). Serological data shows low levels of chikungunya exposure in Senegalese nomadic pastoralists. Pathogens.

[41] Charlier J., Ghebretinsae A., Meyns T., Czaplicki G., Vercruysse J., Claerebout E. (2016). Antibodies against *Dictyocaulus viviparus* major sperm protein in bulk tank milk: Association with clinical appearance, herd management and milk production. Vet. Parasitol..

[42] Deng H., Dam-Deisz C., Luttikholt S., Maas M., Nielen M., Swart A., Vellema P., van der Giessen J., Opsteegh M. (2016). Risk factors related to *Toxoplasma gondii* seroprevalence in indoor-housed Dutch dairy goats. Prev. Vet. Med..

[43] DeCandia A.L., Schrom E.C., Brandell E.E., Stahler D.R., vonHoldt B.M. (2020). Sarcoptic mange severity is associated with reduced genomic variation and evidence of selection in Yellowstone National Park wolves (*Canis lupus*). Evol. Appl..

[44] Klein S.L. (2004). Hormonal and immunological mechanisms mediating sex differences in parasite infection. Parasite Immunol..

[45] Zuk M. (2009). The sicker sex. PLoS Pathog..

[46] Silva P., Galaverni M., Ortega-Del Vecchyo D., Fan Z., Caniglia R., Fabbri E., Randi E., Wayne R.K., Godinho R. (2020). Genomic evidence for the old divergence of Southern European wolf populations. Proc. R. Soc. B.

[47] Rio-Maior H., Nakamura M., Álvares F., Beja P. (2019). Designing the landscape of coexistence: Integrating risk avoidance, habitat selection and functional connectivity to inform large carnivore conservation. Biol. Conserv..

[48] Silva P., López-Bao J.V., Llaneza L., Álvares F., Lopes S., Blanco J.C., Cortés Y., García E., Palacios V., Rio-Maior H. (2018). Cryptic population structure reveals low dispersal in Iberian wolves. Sci. Rep..

[49] Rocha R.G., Magalhães V., López-Bao J.V., Van Der Loo W., Llaneza L., Alvares F., Esteves P.J., Godinho R. (2019). Alternated selection mechanisms maintain adaptive diversity in different demographic scenarios of a large carnivore. BMC Evol. Biol..

[50] Schwensow N., Fietz J., Dausmann K.H., Sommer S. (2007). Neutral versus adaptive genetic variation in parasite resistance: Importance of major histocompatibility complex supertypes in a free-ranging primate. Heredity.

[51] Pimenta V., Barroso I., Álvares F., Correia J., Ferrão da Costa G., Moreira L., Nascimento J., Petrucci-Fonseca F., Roque S., Santos E. (2005). Situação populacional do Lobo em Portugal: Resultados do Censo Nacional 2002/2003.

[52] Boitani L., Ciucci P., Musiani M., Boitani L., Paquet P. (2009). Wolf management across Europe: Species conservation without boundaries. A new Era for Wolves and People: Wolf Recovery, Human Attitudes and Policy.

[53] IPMA Normas Climatológicas 1981–2010. http://www.ipma.pt/pt/oclima/normais.clima/.

[54] INE Instituto Nacional de Estatística-Statistics Portugal. http://www.ine.pt.

[55] Pimenta V., Barroso I., Boitani L., Beja P. (2018). Risks *a la carte*: Modelling the occurrence and intensity of wolf predation on multiple livestock species. Biol. Conserv..

[56] Álvares F., Ferreira C.C., Barbosa A.M., Rosalino L.M., Pedroso N.M., Bencatel J., Bencatel J., Sabino-Marques H., Álvares F., Moura A.E., Barbosa A.M. (2019). Carnívoros. Atlas de Mamíferos de Portugal.

[57] Nakamura M., Godinho R., Rio-Maior H., Roque S., Kaliontzopoulou A., Bernardo J., Castro D., Lopes S., Petrucci-Fonseca F., Álvares F. (2017). Evaluating the predictive power of field variables for species and individual molecular identification on wolf noninvasive samples. Eur. J. Wildl. Res..

[58] Frantz A.C., Pope L.C., Carpenter P.J., Roper T.J., Wilson G.J., Delahay R.J., Burke T. (2003). Reliable microsatellite genotyping of the Eurasian badger (*Meles meles*) using faecal DNA. Mol. Ecol..

[59] Boom R., Sol C.J.A., Salimans M.M.M., Jansen C.L., Wertheim-Van Dillen P.M.E., Van der Noordaa J. (1990). Rapid and simple method for purification of nucleic acids. Cinical Microbiol..

[60] Godinho R., López-Bao J.V., Castro D., Llaneza L., Lopes S., Silva P., Ferrand N. (2015). Real-time assessment of hybridization between wolves and dogs: Combining noninvasive samples with ancestry informative markers. Mol. Ecol. Resour..

[61] Blacket M.J., Robin C., Good R.T., Lee S.F., Miller A.D. (2012). Universal primers for fluorescent labelling of PCR fragments—An efficient and cost-effective approach to genotyping by fluorescence. Mol. Ecol. Resour..

[62] Sikes R.S., Gannon W.L. (2011). Guidelines of the American Society of Mammalogists for the use of wild mammals in research. J. Mammal..

[63] Chinnadurai S.K., Strahl-Heldreth D., Fiorello C.V., Harms C.A. (2016). Best-practice guidelines for field-based surgery and anesthesia of free-ranging wildlife. I. Anesthesia and analgesia. J. Wildl. Dis..

[64] Ferroglio E., Rossi L., Gennero S. (2000). Lung-tissue extract as an alternative to serum for surveillance for brucellosis in chamois. Prev. Vet. Med..

[65] Mörner T., Sandström G., Mattsson R. (1988). Comparison animals of serum and lung extracts for surveys of wild animals for antibodies to *Francisella tularensis* biovar *palaearctica*. J. Wildl. Dis..

[66] Knowles J.E., Frederick C. merTools: Tools for Analyzing Mixed Effect Regression Models. R Package Version 0.2.1. https://cran.r-project.org/package=merTools.

[67] Kuznetsova A., Brockhoff P.B., Christensen R.H. (2017). lmerTest package: Tests in linear mixed effects models. J. Stat. Softw..

[68] R Core Team (2019). R: A Language and Environment for Statistical Computing. https://www.r-project.org/.

[69] Choquet R., Lebreton J.D., Gimenez O., Reboulet A.M., Pradel R. (2009). U-CARE: Utilities for performing goodness of fit tests and manipulating CApture-REcapture data. Ecography.

[70] Genovart M., Pradel R. (2019). Transience effect in capture-recapture studies: The importance of its biological meaning. PLoS ONE.

[71] Burnham K.P., Anderson D.R. (2002). Model. Selection and Multimodel Inference: A Practical Information-Theoretic Approach.

