# Supplementary material for: Non-Invasive Molecular Survey of Sarcoptic Mange in Wildlife: Diagnostic Performance in Wolf Faecal Samples Evaluated by Multi-Event Capture–Recapture Models
Source: Pathogens. 2021 Feb 20;10(2):243. doi: 10.3390/pathogens10020243 (PMC7924033; doi:10.3390/pathogens10020243)
Supplement: Supplementary file 1 [file pathogens-10-00243-s001.pdf]

Supplementary materials

Non-invasive molecular survey of sarcoptic mange in wildlife: diagnostic performance in wolf faecal samples evaluated by multievent capture-recapture models

Julieta Rousseau, Mónia Nakamura, Helena Rio-Maior, Francisco Álvares, Rémi Choquet, Luís Madeira de Carvalho, Raquel Godinho, Nuno Santos

File S1. Methodological approach for multievent capture-recapture model

Assuming the initial state vector *IS*, the survival matrix *S*, the transition matrix *T*, the detection matrix *D*, the test matrix *M* and the event matrix *B* [1]. If π denotes the proportion of newly marked individuals in the state M+ and 1- π in state M-, φ the probability that an individual survives from *t* to *t+1*, ψ the probability that it changes state, *p* the detection probability of an individual, *m* the probability that an individual sample is tested for the presence of *S. scabiei* DNA and *b* the probability that it is assigned a given observation:

S =

	M+	M-	D
M+	Φ	0	1- Φ
M-	0	Φ	1- Φ
D	0	0	1

T =

	M+	M-	D
M+	1- ψ	ψ	0
M-	ψ	1- ψ	0
D	0	0	1

		ND	M+	M-
	M+	1-p	p	0
D =	M-	1-p	0	p
	D	1	0	0

		ND	M+ tested	M- tested	Not tested
	ND	1	0	0	0
M =	M+	0	1- m [(a)]	0	m [(a)]
	M-	0	0	1- m [(a)]	m [(a)]

		0	1	2	3
	ND	1	0	0	0
	M+ tested	0	1-b	b	0
B =	M- tested	0	1- b [(b)]	b [(b)]	0
	Not tested	0	0	0	1

Fixed values in the most supported model:

[(a)] m=0.247 (the proportion of samples not tested for mange)

[(b)] b=0

References:

1. Pradel, R. Multievent: an extension of multistate capture-recapture models to uncertain states. *Biometrics* **2005**, *61*, 442–447, doi:10.1111/j.1541-0420.2005.00318.x.

Table S1. Parametrizations of the most supported model and models with ΔAICc<4. Differences to the most supported model highlighted in bold.

Model parametrization						nP	Deviance	AICc	ΔAICc
#	Survival	Transition between states	Detection	Test for mange	State assignment				
1	Age 1: constant<br>Age 2: constant	Prob. M+ to M- constant<br>Prob. M- to M+ temporal covariate seroprevalence same year	Constant	Prob. not being tested = 0.247	Prob. M- test positive = 0	8	1,099.02	1,115.46	0
2	Age 1: constant<br>**Age 2: mange effect**	Prob. M+ to M- constant<br>Prob. M- to M+ temporal covariate seroprevalence same year	Constant	Prob. not being tested = 0.247	Prob. M- test positive = 0	9	1,097.84	1,116.40	0.94
3	Age 1: constant<br>**Age 2: mange and sex effect**	Prob. M+ to M- constant<br>Prob. M- to M+ temporal covariate seroprevalence same year	Constant	Prob. not being tested = 0.247	Prob. M- test positive = 0	10	1,096.80	1,117.48	2.02
4	Age 1: constant<br>Age 2: constant	Prob. M+ to M- constant<br>Prob. M- to M+ temporal covariate seroprevalence same year	Constant	Prob. not being tested = 0.247	**No constraints**	9	1,099.02	1,117.57	2.11
5	**Age 1: mange effect**<br>**Age 2: mange effect**	Prob. M+ to M- constant<br>Prob. M- to M+ temporal covariate seroprevalence same year	Constant	Prob. not being tested = 0.247	Prob. M- test positive = 0	10	1,097.84	1,118.52	3.06

nP, number of estimable parameters; ΔAICc, difference between the AICc of the current model and that of the most supported model
